# Supplementary material for: A fault-intrusion-tolerant system and deadline-aware algorithm for scheduling scientific workflow in the cloud
Source: PeerJ Comput Sci. 2021 Nov 2;7:e747. doi: 10.7717/peerj-cs.747 (PMC8576554; doi:10.7717/peerj-cs.747)
Supplement: Supplemental Information 6 [file peerj-cs-07-747-s006.zip › Myproject1.1/docs/org/cloudbus/cloudsim/SanStorage.html]

SanStorage


JavaScript is disabled on your browser.


- Overview
- Package
- Class
- Use
- Tree
- Deprecated
- Index
- Help

- Prev Class
- Next Class

- Frames
- No Frames

- All Classes

- Summary:
- Nested |
- Field |
- Constr |
- Method

- Detail:
- Field |
- Constr |
- Method


org.cloudbus.cloudsim

## Class SanStorage

- java.lang.Object
- - org.cloudbus.cloudsim.HarddriveStorage
  - - org.cloudbus.cloudsim.SanStorage

- All Implemented Interfaces:
  :   Storage

  ---

    

  ```
  public class SanStorage
  extends HarddriveStorage
  ```

  SANStorage represents a storage area network composed of a set of harddisks connected in a LAN.
  Capacity of individual disks are abstracted, thus only the overall capacity of the SAN is
  considered. WARNING: This class is not yet fully functional. Effects of network contention are
  not considered in the simulation. So, time for file transfer is underestimated in the presence of
  high network load.

  Since:
  :   CloudSim Toolkit 1.0

- - ### Constructor Summary

    Constructors

    Constructor and Description

    `SanStorage(double capacity,
    double bandwidth,
    double networkLatency)`

    Creates a new SAN with a given capacity, latency, and bandwidth of the network connection.

    `SanStorage(java.lang.String name,
    double capacity,
    double bandwidth,
    double networkLatency)`

    Creates a new SAN with a given capacity, latency, and bandwidth of the network connection.
  - ### Method Summary

    Methods

    | Modifier and Type | Method and Description |
    | --- | --- |
    | `double` | `addFile(File file)` Adds a file to the storage. |
    | `double` | `addFile(java.util.List<File> list)` Adds a set of files to the storage. |
    | `double` | `addReservedFile(File file)` Adds a file for which the space has already been reserved. |
    | `double` | `deleteFile(File file)` Removes a file from the storage. |
    | `double` | `deleteFile(java.lang.String fileName, File file)` Removes a file from the storage. |
    | `double` | `getMaxTransferRate()` Gets the maximum transfer rate of the storage in MB/sec. |

    - ### Methods inherited from class org.cloudbus.cloudsim.HarddriveStorage

      `contains, contains, deleteFile, getAvailableSpace, getAvgSeekTime, getCapacity, getCurrentSize, getFile, getFileNameList, getLatency, getName, getNumStoredFile, hasPotentialAvailableSpace, isFull, renameFile, reserveSpace, setAvgSeekTime, setAvgSeekTime, setLatency, setMaxTransferRate`
    - ### Methods inherited from class java.lang.Object

      `clone, equals, finalize, getClass, hashCode, notify, notifyAll, toString, wait, wait, wait`

- - ### Constructor Detail


    - #### SanStorage

      ```
      public SanStorage(double capacity,
                double bandwidth,
                double networkLatency)
                 throws ParameterException
      ```

      Creates a new SAN with a given capacity, latency, and bandwidth of the network connection.

      Parameters:
      :   `capacity` - Storage device capacity
      :   `bandwidth` - Network bandwidth
      :   `networkLatency` - Network latency

      Throws:
      :   `ParameterException` - when the name and the capacity are not valid


    - #### SanStorage

      ```
      public SanStorage(java.lang.String name,
                double capacity,
                double bandwidth,
                double networkLatency)
                 throws ParameterException
      ```

      Creates a new SAN with a given capacity, latency, and bandwidth of the network connection.

      Parameters:
      :   `name` - the name of the new harddrive storage
      :   `capacity` - Storage device capacity
      :   `bandwidth` - Network bandwidth
      :   `networkLatency` - Network latency

      Throws:
      :   `ParameterException` - when the name and the capacity are not valid
  - ### Method Detail


    - #### addReservedFile

      ```
      public double addReservedFile(File file)
      ```

      Adds a file for which the space has already been reserved.

      **Specified by:**
      :   `addReservedFile` in interface `Storage`

      **Overrides:**
      :   `addReservedFile` in class `HarddriveStorage`

      Parameters:
      :   `file` - the file to be added

      Returns:
      :   the time (in seconds) required to add the file


    - #### getMaxTransferRate

      ```
      public double getMaxTransferRate()
      ```

      Gets the maximum transfer rate of the storage in MB/sec.

      **Specified by:**
      :   `getMaxTransferRate` in interface `Storage`

      **Overrides:**
      :   `getMaxTransferRate` in class `HarddriveStorage`

      Returns:
      :   the maximum transfer rate in MB/sec


    - #### addFile

      ```
      public double addFile(File file)
      ```

      Adds a file to the storage.

      **Specified by:**
      :   `addFile` in interface `Storage`

      **Overrides:**
      :   `addFile` in class `HarddriveStorage`

      Parameters:
      :   `file` - the file to be added

      Returns:
      :   the time taken (in seconds) for adding the specified file


    - #### addFile

      ```
      public double addFile(java.util.List<File> list)
      ```

      Adds a set of files to the storage. Runs through the list of files and save all of them. The
      time taken (in seconds) for adding each file can also be found using
      `gridsim.datagrid.File#getTransactionTime()`.

      **Specified by:**
      :   `addFile` in interface `Storage`

      **Overrides:**
      :   `addFile` in class `HarddriveStorage`

      Parameters:
      :   `list` - the files to be added

      Returns:
      :   the time taken (in seconds) for adding the specified files


    - #### deleteFile

      ```
      public double deleteFile(java.lang.String fileName,
                      File file)
      ```

      Removes a file from the storage. The time taken (in seconds) for deleting the file can also
      be found using `gridsim.datagrid.File#getTransactionTime()`.

      **Specified by:**
      :   `deleteFile` in interface `Storage`

      **Overrides:**
      :   `deleteFile` in class `HarddriveStorage`

      Parameters:
      :   `fileName` - the name of the file to be removed
      :   `file` - the file which is removed from the storage is returned through this parameter

      Returns:
      :   the time taken (in seconds) for deleting the specified file


    - #### deleteFile

      ```
      public double deleteFile(File file)
      ```

      Removes a file from the storage. The time taken (in seconds) for deleting the file can also
      be found using `gridsim.datagrid.File#getTransactionTime()`.

      **Specified by:**
      :   `deleteFile` in interface `Storage`

      **Overrides:**
      :   `deleteFile` in class `HarddriveStorage`

      Parameters:
      :   `file` - the file which is removed from the storage is returned through this parameter

      Returns:
      :   the time taken (in seconds) for deleting the specified file


- Overview
- Package
- Class
- Use
- Tree
- Deprecated
- Index
- Help

- Prev Class
- Next Class

- Frames
- No Frames

- All Classes

- Summary:
- Nested |
- Field |
- Constr |
- Method

- Detail:
- Field |
- Constr |
- Method
